# Experimental and statistical study of the effect of temperature and waste ratio on the mechanical properties and cost of polystyrene polypropylene plastic blends

**DOI:** 10.1016/j.heliyon.2020.e04166

**Published:** 2020-06-10

**Authors:** Saeed Sameer Ibrahim Almishal, Tamer A. Mohamed, Mostafa Shazly

**Affiliations:** Mechanical Engineering Department, Faculty of Engineering, The British University in Egypt, Suez Desert Road, P.O. Box 43, El Sherouk City, Cairo, 11837, Egypt

**Keywords:** Mechanical engineering, Materials characterization, Manufacturing engineering, Polymers, Mechanical property, Waste treatment, Plastic blends, Recycling, ANOVA, Polystyrene, Polypropylene, Waste ratio

## Abstract

Since most plastics are not biodegradable, plastic recycling is the main part of global efforts to reduce plastic in the waste stream. Sorting of plastics imposes lots of difficulties which can be avoided by introducing plastic blends. This paper starts by reviewing the recent attempts to study plastic blends. Accordingly, the purpose of this study is to analyze experimental results and apply statistical measures using ANOVA to study the effect of increasing the waste ratio that contains both waste polystyrene and polypropylene on the mechanical properties of pure polystyrene when injected at different temperatures. Cost is taken as a response factor to analyze whether the degradation of mechanical properties is justified by a decrease in cost. As expected, cost dramatically decreases with increasing the waste ratio at any temperature. Increasing the waste ratio resulted in better mechanical properties with a maximum at a 30% waste ratio at 200 °C and 220 °C. This paper ends with a multi-objective optimization analysis that helps decision-makers optimize the properties needed of the studied plastic blend by controlling both the temperature and waste ratio.

## 1 Introduction

Recovered plastic wastes must be sorted and cleaned before reforming. Sorting is one of the most critical stages in the plastic recycling as it could affect the quality of the whole process. Sorting focuses on identifying the plastic materials from the mixed wastes and differentiating between the different types of plastics to be separated. Indeed, sorting can be done manually or automatically. Sorting is then followed by cleaning which takes into consideration the extent of contamination, the contaminating chemicals, and the intended use of the end-product. Aqueous solutions can be used to remove surface contaminants (Manrich and Santos, 2009). These recovery stages are important as mixed plastics have poor properties due to the inhomogeneous melting and burning actions which result in poor adhesion and separate phases with weak interfaces. This behavior can be described thermodynamically by the low entropy of mixing. Accordingly, almost identical-composition resins that yield high entropy of mixing should be utilized. Unfortunately, same-type postconsumer plastics recycling is limited by the technological advancements in sorting. However, some applications based on heterogonous blends such as plastic lumber are being considered (Kutz, 2011; Masters and Ela, 1991). Plastics must be sorted carefully by type, color, shape and texture. Different dry and wet separating techniques have been developed for sorting but each with its complexities and limitation. Not to mention, there are thousands of variations of polymers and most separating techniques available can only separate one or two types of plastics from the group of waste plastics. For instance, it is difficult to separate shredded bottles of PVC from PET. Dodbiba and Fujita (2004) reviewed different techniques that had been developed since 1970 to separate PVC from PET including Tri-Flo separator, LARCODEMS separator, TACUB jig, drum separator, zigzag air classifier, air table, fluidized bed triboelectric cyclone separator, rotating drum and others. Indeed, each method has its limited efficiency to separate the flakes of PVC from PET (Dodbiba and Fujita, 2004). Clear PET and unpigmented HDPE milk bottles can be identified by Fourier-transform near-infrared (FT-NIR) spectroscopy with the use of optical color recognition camera. Multiple optical sensors can sort clear PET containers from colored ones. Polyolefins can be separated from PVC, PET and Polystyrene (PS) by sink/float separation in water. Laser sorting systems can also be applied for sorting purposes. The flexible packaging is the most difficult to sort due to deficiencies in their related sorting equipment (Hopewell et al., 2009). While integrated sorting techniques can be promising for separating different plastics, they still need a huge amount of research and improvement to reduce their cost, size and complexity.

Sorting and its complexities can be avoided by mixing plastics and optimizing the properties of the mix. Mixing presents a much easier and more economical solution than sorting. Mixing existing plastics results in plastic alloys and blends. A plastic alloy has a definite glass transition temperature in contrary to plastic blends. Blends are more like composites where properties are compromised between the constituents while plastic alloys usually show better properties than their constituents (Goodship, 2017). The compatibility and adhesion between different types of polymers is extremely limited which prevents the formation of plastic blends with good mechanical properties. For example, adding Polypropylene (PP) as a minor in a major PS matrix is claimed to result in a plastic blend that has zero compatibility and no adhesion between its two constituents. Good adhesion can only be found between polymers of the same type (Kutz, 2011; Rosato et al., 2010).

Wei et al. (2016) reported that increasing the percentage of recycled HDPE decreased the tensile strength of the blend but increased the ductility. The study also reported the effect of the calcium carbonate filler on the mechanical properties and found that increasing its content, in general, deteriorated these properties. However, a compromise and optimization are achieved for a reduced cost by 20% recycled plastic and 0.1% of calcium carbonate. Similar results were achieved by Shi et al. (2013) on Polypropylene (PP). They went even far by proposing that half/half mix of virgin and recycle PP can be an efficient and perfect choice for reinforcing concrete.

Two adopted approaches for improving the behavior of plastic blends are additives and intrinsic optimization. The additive approach depends on utilizing chemical agents such as compatibilizers, stabilizers, fillers, impact modifiers, and smoke suppressors (LaMantia, 2001). The second approach relies on achieving optimization percentages between virgin and recycled plastics. Optimization is also achieved for processing parameters and machine components. This can be achieved by the design and analysis of experiments integrated with Taguchi optimization methods (Gu et al., 2014).

The Flory−Huggins interaction parameter, χ, describes the binary interactions of polymer blends. It is typically reported as a function of temperature, with temperature-independent contribution χ_S_ of entropic origin that is added to enthalpic term; χ_H_/T. For polystyrene and polypropylene, χ is often dominated by the enthalpic term due to the mismatch between different monomers in cohesive energy density. Generally, larger χ value results from the large mismatch in cohesive energy density which accordingly results in a greater driving force for demixing (Bates and Fredrickson, 1990). Therefore, the PS/PP blend is immiscible and incompatible. This can be further elaborated by the fact that PS contains aromatic benzene rings, while PP contains straight carbon chains of an aliphatic kind. Some researchers have reported on using styrene-b-(ethylene-cobutylenes)-b-styrene (SEBS) as a compatibilizer in the optimization of immiscible PS/PP blends (Kamal et al., 1997; Radonjič, 1999; Tambasco et al., 2006).

Chemical degradation is the main factor when the same material is recycled multiple times or even a single time. Plastics behave differently to recycling, for instance, PP does not get affected by recycling or impurities as polystyrene. Another example is HDPE which shows almost constant tensile strength regardless of recycling runs (Takatori, 2015). The ease of material to fill the mold is measured by the melt flow index measured by g/10min. The behavior is irregular and shows that PP keeps moving around the mean of its properties. This can be explained by the entanglement formation in compensation of bonds breakage (Weckström, 2012).

Very little investigations were carried on understanding the behavior of plastic blends and waste-plastic blends along with their mechanical properties. Thus, the current work investigates the viability of plastic blends as a method or a solution for plastic recycling without the need for sorting. This paper statistically studies the controllable factors that directly affect the performance of the uncompatibilized plastic blends that constitute raw and waste plastics namely; waste ratio and injection temperature. The performance is characterized by a set of mechanical properties along with cost analysis. Moreover, it is intended to develop an optimization function that balances between the different responses.

## 2 Experimental preparation and methodology

This section explains in details the materials that will be used in the experiment and the method and technique of preparing the test specimens that will be used to perform the experiments.

### 2.1 Materials

**Figure 1:**
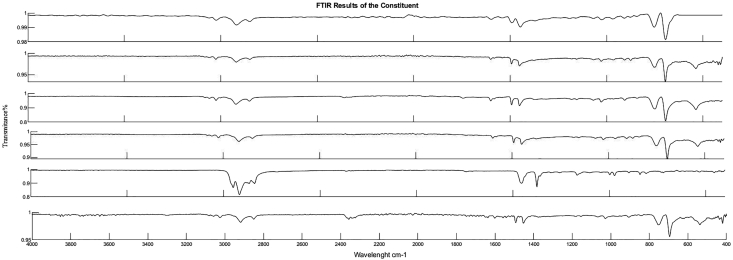
FTIR test results.

The plastic blends in this study are based on pure polystyrene. Plastic wastes were gathered from five different sources, that include plastic products factories and hotels, and were crushed to small sizes, with a screen size of 12mm. The different plastic waste batches were identified by performing FTIR testing. Four of the batches appeared to be waste polystyrene and one batch appeared to be waste polypropylene. The four waste polystyrene batches are from different sources and have different colors. The results of the FTIR testing are reported in Figure 1. Starting from the top; the four PS waste batches followed by the PP waste batch then the pure PS. The waste mixture is prepared based on mass to contain 80% recycled PS and 20% recycled PP. This 80% recycled PS constitutes equal percentages from each of the four sources of recycled PS.

### 2.2 Specimens preparation and testing

All the crushed plastics and the pure polystyrene are dehumidified first, at 80 °C for 20 min, and then ground into two stages using two different grinders to ensure smooth grind. Appropriate mixtures of 20% and 50% waste content are prepared and mixed through the grinder, with a screen size of 3–5 mm. The mixtures are then injected using vertical plunger plastic injection molding machine at different temperatures while holding the residence time in the barrel and injection pressure constant. The machine used is a benchtop injection molding machine with air compressor Model WZ30000 with Vertex VT4826 Fuzzy Enhanced PID Temperature Controller. Table A1, Appendix A, shows a photo of the machine and its specifications (Kunlun, 2014). The molten mixture is injected into a mold to take the form of standard dumbbell-shaped test specimens according to the Standard Test Method for Tensile Properties of Plastics ASTM-D638. The shape and dimensions of the specimens are given in Table A2 (ASTM International, 2014). The specimens are weighed on a sensitive electronic balance with an accuracy of 0.005 g and then are tested using at a universal testing machine at room temperature and a strain rate of 1 mm/min.

## 3 The factorial design

In this study, the effect of increasing the waste ratio on the properties of pure polystyrene when injected at different temperatures on four responses namely, ultimate tensile strength (UTS), toughness, stiffness, and cost are studied. The controllable factors are injection temperature and waste ratio. The injection temperature is one of the most critical parameters to monitor. Low injection temperature results in improper mixing and thus lowers the homogeneity of the blend, while high injection temperatures may lead to burns and severe weakening of the structure. The temperature range of 180 °C–220 °C is chosen based on the appropriate temperature range of injecting pure polystyrene. The effect of increasing the waste ratio in the blend on four responses is investigated as well. Consequently, both temperature and waste ratios are varied at only three levels as the experiments are time-consuming. Accordingly, the experiment is carried at three levels of the waste mass ratio which are namely; 0%, 20% and 50% at temperatures of 180 °C, 200 °C, and 220 °C.

Response factors in the present study are divided into technical and economical response factors. As for the technical response factors, the stress-strain curve is generated for each specimen and three response factors are analyzed. The first one is the ultimate tensile strength which is the maximum stress of the stress-strain curve and a measure of the strength of the blend. The second one is toughness which is a measure of the energy of the deformation and is found from the area under the stress-strain curve. The third one is the stiffness which measures the resistance to deformation and calculated from the slope of the curve.

As for the economical response factor, the cost of the blend is considered. The cost is an extremely vital factor that needs to be deliberately investigated. Considering cost relates the abstract experiment to the production and industry. The cost has two components which are mainly the materials cost and the energy cost. The cost is affected by the resultant density of the blend which increases the complexity of its analysis. Considering the three technical factors and the cost generates and covers the full picture and features of the production of the plastic blend.

## 4 Results and discussion

The experiment has four replicates for each combination to increase the reliability of the model. The results of the experiment are analyzed in this section. For each response factor, the analysis of variance (ANOVA) in its reduced model, contour plot and response surface, and the regression model of the results are presented and discussed. It is worth saying that for each response factor, the residual plot is tested to ensure that the ANOVA assumptions are valid and justified.

### 4.1 Ultimate tensile strength (UTS)

The reduced ANOVA table for the strength is shown in Table A3. The model F-value of 25.65 implies the model is significant. There is only a 0.01% chance that an F-value this large could occur due to noise. P-values less than 0.05 indicate model terms are significant. In this case temperature, waste ratio and their first order interaction are significant model terms. Moreover, the square of waste ratio and the interaction between the square of temperature and waste ratio are significant as well. The lack of fit F-value of 1.38 implies that it is not significant relative to the pure error. There is a 24.96% chance that a Lack of Fit F-value this large could occur due to noise. The determination coefficient (R^2^) is found to be 0.8651 which supports the acceptance of the model. The adequate precision ratio of 16.969 is obtained which indicates an adequate signal and that the model can be used to navigate the design space. It can also be concluded that the model does not contain too many insignificant factors due to the reasonable agreement between Predicted and Adjusted R^2^, 0.7708 and 0.8314 respectively.

The adequacy of the strength model is tested from the residual plots. Figure B1, Appendix B, shows that the residuals are normally distributed. Figure B2 shows the random distribution of the residuals and Figure B3 shows the almost constant variance behavior of the residuals. These three results confirm the adequacy of the ANOVA analysis. The same check tests are carried for the other response factors, but they are not presented in the paper for simplicity.

The equation in terms of temperature and waste ratios can be used to make predictions about the strength for given levels of each factor as illustrated in Eq. (1). Indeed, the levels should be specified in the original units for each factor. The temperature in Celsius and waste ratio as the numeric value of the percentage (e.g. 0, 20, 50 ...).

(1) UTS (MPa) = +416.66662–3.65835T - 13.66571W + 0.126518 T∗W + 0.008275 T^2^ + 0.022021W ^2^ - 0.000286 T^2^∗W - 0.000124T∗W^2^

**Figure 2:**
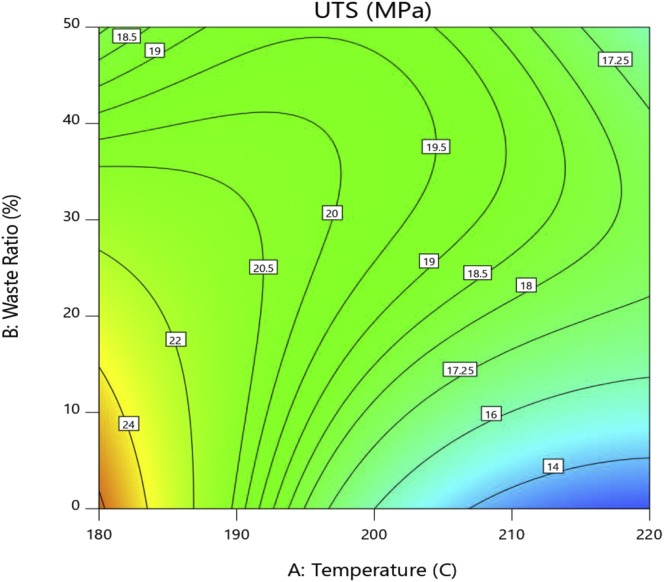
Contour surface for UTS

The surface contour plot, shown in Figure 2, gives insight on how to optimize the strength by changing the temperature and waste ratio. Although the behavior of the response is complex, it can be observed that strength dramatically decreases with increasing injection temperature of the pure PS. This behavior is assuaged for the 20% waste ratio mixture. However, the 50% waste ratio shows a different behavior of optimum strength at 200 °C injection temperature that decreases in either direction. Increasing the waste ratio at a constant injection temperature of 180 °C decreases the strength significantly. This behavior is shifted to increase in strength at 200 °C whereas, at 220 °C, it is predicted that a mixture with 30% waste ratio will be optimum.

### 4.2 Toughness

Table A4 shows the reduced ANOVA for toughness and summarizes the P-values figures. The toughness model F-value is 16.48 which implies the model is significant. In other words, there is only a 0.01% chance that this large F-value occurred due to noise. The toughness is not impervious to temperature, waste ratio, and their interaction. Moreover, the square of temperature, the interaction between the square of temperature and waste ratio and the interaction between the square of temperature and square of the waste ratio are significant model terms. The determination coefficient (R^2^) is found to be 0.8300 which supports the acceptance of the model. The predicted R^2^ of 0.6978 is in reasonable agreement with the adjusted R^2^ of 0.7796. The adequate precision ratio is 13.628 which indicates an adequate signal and that the model can be used to navigate the design space.

The regression equation in terms of temperature and waste ratios can be used to make predictions about the toughness for given levels of each factor as illustrated in Eq. (2). The final result of toughness is given by Joules.

(2) Toughness = +26.55124–0.238543T + 1.88965W - 0.018925T∗W +0.000545 T^2^ - 0.061706W ^2^ + 0.000048T^2^∗W +0.000614T∗W^2^ - 1.53729E - 06T^2^∗W^2^

**Figure 3:**
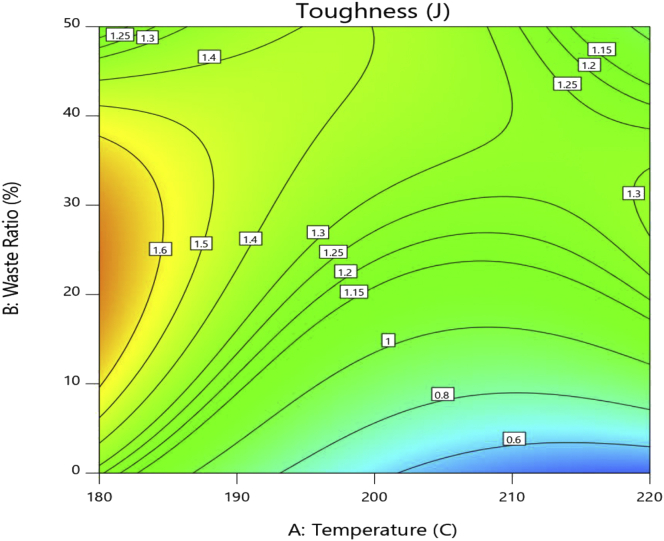
Toughness contour plot.

The surface contour, shown in Figure 3, helps in understanding the complex behavior of toughness and on how to optimize it by changing the controllable factors; temperature and waste ratio. It can be observed that toughness dramatically decreases with increasing injection temperature of the pure PS. While for the 20% waste ratio mixture there is a single minimum at 200 °C. On the contrary, this minimum shifts to a maximum for the 50% waste ratio at the same temperature of 200 °C. Maintaining injection temperature at 180 °C while increasing the waste ratio shows a maximum at approximately 20% waste ratio with a decrease in toughness otherwise. The same behavior is observed at 220 °C but the maximum shifts to roughly 30% waste ratio. Eventually, increasing the waste ratio at a constant injection temperature of 200 °C enlarges the toughness significantly. This can be attributed to the fact that increasing the waste mixture ration increases the percentage of PP which has much higher toughness and elongation than PS. In addition, better mixing and homogeneity results generally at 200 °C due to the decreased viscosity of the mix.

### 4.3 Stiffness

The reduced ANOVA table for stiffness is shown in Table A5. The stiffness model F-value is18.39 corresponding to p-value < 0.0001 indicating a significant model. For the stiffness case, many high order interactions are significant. The predicted R^2^ of 0.7244 is in reasonable agreement with the adjusted R^2^ of 0.7990. The adequate precision is 12.621 implying an adequate signal and that the model can be used to navigate the design space. The determination factor R^2^ is found to be 0.8450 which support the significance of the model and that the variability presented in the behavior of stiffness is well captured.

The regression equation for the stiffness in terms of temperature and waste ratios can be used to make predictions about the stiffness for given levels of each factor as illustrated in Eq. (3).

(3) Stiffness (MPa) = +21060.41214–193.67311T - 2191.28905W + 21.64483T∗W + 0.453648T ^2^ + 32.10615W^2^ - 0.052847T^2^∗W - 0.317655T∗W^2^ +0.000777T ^2^∗W^2^

**Figure 4:**
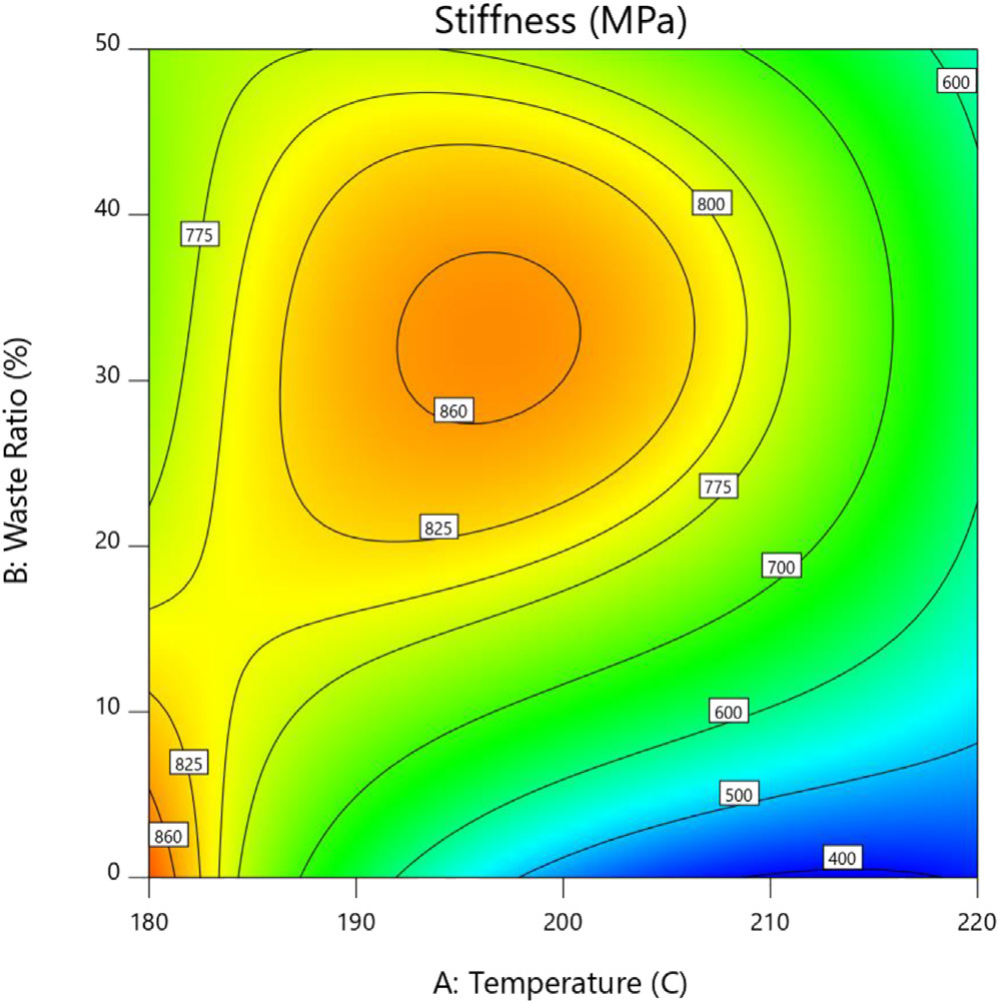
Stiffness contour plot.

The surface contour plot, shown in Figure 4, demonstrates the convoluted behavior of stiffness and on how it can be optimized. Stiffness dramatically decreases with increasing injection temperature of the pure PS however this drop decelerates at the vicinity of 220 °C. While for the 20% and 50% waste ratios, stiffness reaches a maximum at roughly 195 °C. Sustaining injection temperature at 180 °C while increasing the waste ratio leads to a slow and not that significant decrease in stiffness. This can be attributed to the fact that stiffness of both PP and PS are roughly of the same magnitude. However, at 200 °C there is an evident sharp increase up to the maximum at roughly 30% waste ratio. The same occurs at 220 °C but the increase is tempered, which can be explained by the improved homogeneity of the structure at higher processing temperatures.

### 4.4 Cost

This is the fourth and last response factor to be analyzed in our experiment to check the feasibility of the experimental work and ensure that the best solution is practically and economically feasible and justify the choice of the levels of the controllable factors. The reduced ANOVA table for the cost is shown in Table A6. The cost model F-value of 130.34 supports the fact that the model is significant with only a 0.01% chance that this large F-value is due to noise. Although injection temperature is not significant for cost, its square value term A^2^ is significant. Waste ratio and its square value term along with the interaction between the square of temperature and the square of the waste ratio are significant model terms. The predicted R^2^ of 0.9567 is in reasonable agreement with the adjusted R^2^ of 0.9682 and the adequate precision is 29.084; indicating a satisfactory signal and the model can be used to navigate the design space. The determination factor (R^2^) is calculated to be 0.9757 which implies a very strong correlation.

The equation in terms of temperatures and waste ratios can be used to make predictions about the cost as well for given levels of each factor as illustrated in Eq. (4). The result of this equation represents the cost of 1 dm^3^ volume in Egyptian Pounds.

(4) Cost = -285.94918 + 4.16915T + 119.03455W - 1.20370T∗W - 0.010431T^2^ - 2.37251W^2^ +0.002993T ^2^∗W +0.023650T∗W^2^ - 0.000059T^2^∗W^2^

**Figure 5:**
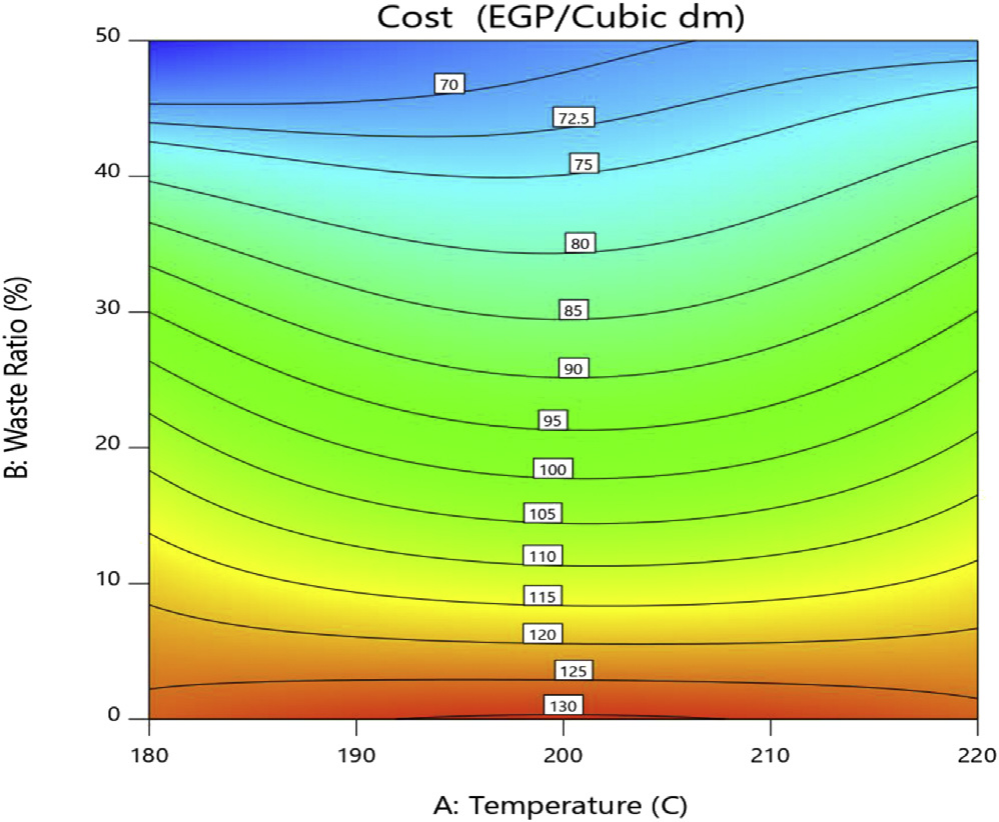
Cost contour plot.

The surface contour plot, shown in Figure 5, demonstrates the behavior of the cost and how it can be minimized. Cost dramatically decreases with increasing the waste ratio. For the 50% waste ratio, there is a small increase that can be observed in cost with the increase in injection temperature justified by the increase in energy. At 200 °C injection temperature, the pure PS shows a maximum while the 50% waste ratio shows a minimum of cost. This inconsistent behavior could be explained by the increase in the required energy and the variation in both the melt flow index and the resulted blend density.

## 5 Multi-objective optimization

In the previous section, each of the four responses were analyzed separately. However, the decision-maker will attempt to optimize all the responses simultaneously according to specified criteria by selecting the controllable parameters namely, injection temperature and waste ratio. This is performed using the Optimization module of the Design-Expert 11.1.0 software. This module solves the regression models presented previously for a combination of controllable factor values that simultaneously meet the requirements placed on each of the responses. The technical response factors must be maximized, and the cost must be minimized. The importance of each response can be changed in relation to other responses. Three levels of importance are chosen for this study; (∗) represents low importance while (∗∗∗) represents medium importance and (∗∗∗∗∗) represents high importance. To give more emphasis to the goal weight of 10 is chosen for all the responses which accordingly adjust the desirability function in which the tool seeks to optimize. This results in 81 cases solved and documented in Table 1 below. In each cell, the first number represents the solved injection temperature and the second number represents the solved percentage of waste ratio corresponding to the importance given for each of the responses. For instance, if the decision makers are mainly concerned with optimizing strength for their product but they are least concerned with toughness, and at the same time minimizing cost and optimizing stiffness are both of medium importance, they should select the grey colored cell in Table 1. This cell corresponds to (∗∗∗∗∗) strength, (∗∗∗) cost, (∗∗∗) stiffness and (∗) toughness. Which means they should prepare a 39.3% waste blend and inject it at 193.870 °C.

**Table 1:** Decision maker optimization matrix.

Stiffness	Strength	Toughness								
		∗			∗∗∗			∗∗∗∗∗		
		Cost								
		∗	∗∗∗	∗∗∗∗∗	∗	∗∗∗	∗∗∗∗∗	∗	∗∗∗	∗∗∗∗∗
∗	∗	193.079–40.346	192.017–48.029	188.172–50.000	180.000–28.024	193.063–45.861	192.401–49.617	180.000–26.449	192.067–44.111	193.471–48.153
	∗∗∗	180.000–19.822	193.568–44.371	191.973–48.969	180.000–21.703	192.630–43.273	193.248–47.120	180.000–22.3570	180.000–31.933	193.306–46.147
	∗∗∗∗∗	180.000–11.866	191.972–48.968	193.424–46.064	180.000–16.822	191.230–40.495	193.348–45.211	180.000–18.884	180.000–28.495	192.843–44.332
**∗∗∗**	∗	194.556–36.923	194.770–41.577	193.275–45.380	191.398–36.584	193.510–41.663	193.337–44.698	180.000–23.834	192.193–41.094	193.048–44.151
	∗∗∗	193.015–35.532	194.407–40.417	194.094–43.590	180.000–17.300	193.074–40.347	193.561–43.354	180.000–19.537	191.307–39.440	192.967–42.951
	∗∗∗∗∗	180.000–5.206	193.870–39.336	194.159–42.396	180.000–12.735	192.350–38.998	193.468–42.238	180.000–16.094	189.197–36.786	192.687–41.824
∗∗∗∗∗	∗	195.167–35.602	195.485–39.064	195.102–41.725	193.174–36.025	194.226–39.508	194.270–41.960	190.357–35.038	193.020–39.494	193.567–41.932
	∗∗∗	194.071–34.938	194.937–38.483	194.938–41.005	180.000–12.378	193.734–38.734	194.127–41.183	180.000–16.305	192.425–38.530	193.367–41.124
	∗∗∗∗∗	180.000–0.000	194.368–37.853	194.679–40.353	180.000–8.631	193.136–37.908	193.878–40.468	180.000–13.112	191.599–37.389	193.073–40.342

## 6 Conclusion

Plastic blends offer an easier and more effective solution for plastic recycling than of sorting. The study of polystyrene-polypropylene waste plastic blends revealed that pure polystyrene is highly favored only when both optimization of strength and stiffness are of high importance. However, when cost and toughness are brought to the equation; waste plastic blends showed better results. The ideal injection temperature and waste ratio that guarantee certain optimization criteria cannot be found easily. For instance, a waste ratio of 40.34% injected at 193 °C provided an optimization with equal importance for all of the response factors.

This is mainly due to the fact that the four response factors behave differently when changing temperature and waste ratio. All the mechanical properties dramatically decreases with increasing injection temperature of the pure PS. For the 20% waste ratio mixture, while strength keeps decreasing with temperature, toughness reached a minimum at 200 °C and stiffness reached a maximum at roughly 195 °C. For the 50% waste ratio, each of strength and toughness had a maximum value at 200 °C injection temperature. This maximum point slightly shifted to 195 °C for stiffness. At the same ratio, there was a small increase in cost due to energy requirements.

Increasing the waste ratio at a constant injection temperature of 180 °C did not significantly affect stiffness but it led to a significant decrease in the strength and a maximum was observed at a 20% waste ratio for toughness. For increasing the waste ratio at 200 °C and 220 °C led generally to better mechanical properties that were optimized at a 30% waste ratio mixture. Cost dramatically decreased with increasing the waste ratio at any temperature. Despite the new and rising efforts to understand and characterize plastic blends, more research is needed to develop models that cover all range of plastics and properties that can consequently provide ultimate optimization tools for non-sorting plastic recycling.

## Declarations

### Author contribution statement

Saeed Sameer Ibrahim Almishal: Conceived and designed the experiments; Performed the experiments; Analyzed and interpreted the data; Contributed reagents, materials, analysis tools or data; Wrote the paper.

Tamer A. Mohamed & Mostafa Shazly: Conceived and designed the experiments; Analyzed and interpreted the data; Wrote the paper.

### Funding statement

This research did not receive any specific grant from funding agencies in the public, commercial, or not-for-profit sectors.

### Competing interest statement

The authors declare no conflict of interest.

### Additional information

No additional information is available for this paper.

## Appendix A Supplementary data

APPENDIX A-B.docx

## References

[bib1] ASTM D638-14 (2014). Standard Test Method for Tensile Properties of Plastics. http://www.astm.org.

[bib2] Bates F.S., Fredrickson G.H. (1990). Block copolymer thermodynamics: theory and experiment. Annu. Rev. Phys. Chem..

[bib3] Dodbiba G., Fujita T. (2004). Progress in separating plastic materials for recycling. Phys. Sep. Sci. Eng..

[bib4] Dongguan Kunlun Testing Instrument (2014). Benchtop injection molding machine. http://www.kunluninstrument.com/ProductShow_159.html.

[bib5] Gu Fu, Hall P., Miles N.J., Ding Qianwen, Wu Tao (2014). Improvement of mechanical properties of recycled plastic blends via optimizing processing parameters using the Taguchi method and principal component analysis. Mater. Des..

[bib6] Goodship V. (2017). Arburg Practical Guide Injection Moulding.

[bib7] Hopewell J., Dvorak R., Kosior E. (2009). Plastics recycling: challenges and opportunities. Philos. Trans. R. Soc. London, Ser. A B.

[bib8] Kamal M.R., Demarquette N.R., Lai-Fook R.A., Price T.A. (1997). Evaluation of thermodynamic theories to predict interfacial tension between polystyrene and polypropylene melts. Polym. Eng. Sci..

[bib9] Kutz M. (2011). Applied Plastics Engineering Handbook.

[bib10] LaMantia F. (2001). Recycling of Plastic Materials.

[bib11] Manrich S., Santos A.S.F. (2009). Plastic Recycling. [electronic Resource].

[bib12] Masters G.M., Ela W.P. (1991).

[bib13] Radonjič G. (1999). Compatibilization effects of styrenic/rubber block copolymers in polypropylene/polystyrene blends. J. Appl. Polym. Sci..

[bib14] Rosato D., Rosato M., Schott N. (2010).

[bib15] Shi Y., Tuladhar R., Combe M., Collister T., Jacob M., Shanks R., Dvorsky A.T., Kohoutkova A., Vytlacilova V. (2013). Mechanical properties of recycled plastic fibres for reinforcing concrete. Proceedings of 7th International Conference Fibre Concrete 2013, Prague, Czech Republic, 12-13 September 2013.

[bib16] Takatori E. (2015). Material recycling of polymer materials & material properties of the recycled materials. Int. Pol. Sci. Tech..

[bib17] Tambasco M., Lipson J.E.G., Higgins J.S. (2006). Blend miscibility and the Flory− huggins interaction parameter: a critical examination. Macromolecules.

[bib18] Weckström D. (2012). Changes in Mechanical Properties of Recycled Polypropylene.

[bib19] Wei X., Liu W., Zhang M., Gao X. (2016). Study on the identification of adulteration of polycarbonate drinking bottles with postconsumer recycled plastics. Mater. Sci. Forum.

