# Supplementary material for: Experimental and statistical study of the effect of temperature and waste ratio on the mechanical properties and cost of polystyrene polypropylene plastic blends
Source: Heliyon. 2020 Jun 10;6(6):e04166. doi: 10.1016/j.heliyon.2020.e04166 (PMC7292924; doi:10.1016/j.heliyon.2020.e04166)
Supplement: Supplementary file 1 — APPENDIX A-B.docx [file mmc1.docx]

**APPENDIX A**

**Table A1. WZ30000 Injection Machine**

| Item | Specification |  |
| --- | --- | --- |
| Maxi. temperature | 490℃ | 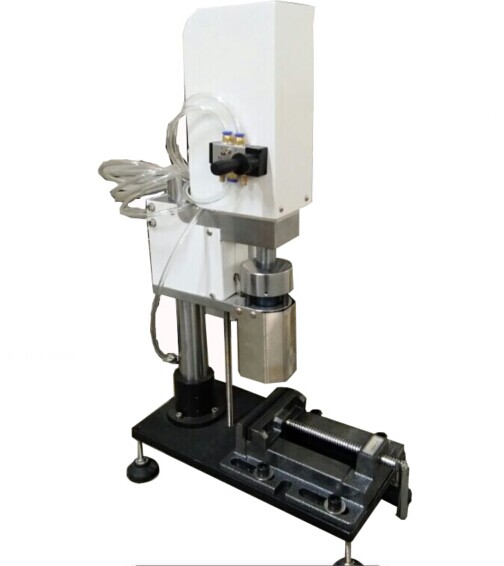 |
| Plunger diameter | 30 mm |  |
| Maximum compressing stoke | 135 mm |  |
| Heating cavity size | 30*70 mm |  |
| Maximum shot volume | 30cm^3^(20g) |  |
| Mold max. size | 120*120*80 mm |  |
| Machine weight | 50 kg |  |
| Power supply | 400 W |  |
| Voltage | 220V/230V, 50/60HZ |  |

**Table A2. Dimensions and Shape of the Testing Specimens**

| Item | Dimension |  |
| --- | --- | --- |
| Thickness, T | 4 mm | 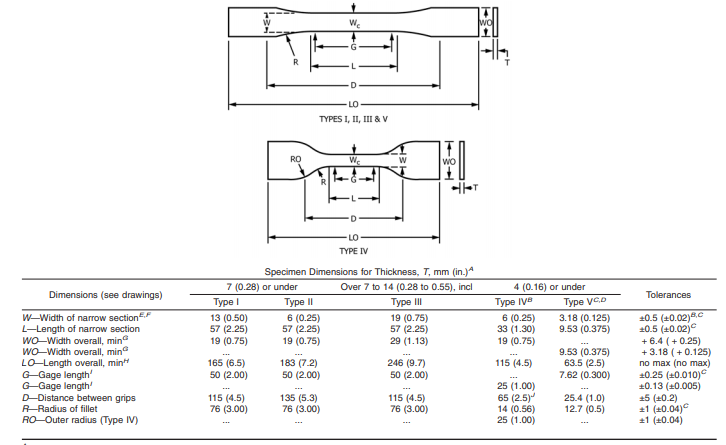 |
| Width of Narrow Section, W | 13 mm |  |
| Length of Narrow Section, L | 57 mm |  |
| Width Overall, WO | 19 mm |  |
| Length of Overall, LO | 165 mm |  |
| Gage length, G | 50 mm |  |
| Distance between grips, D | 115 mm |  |
| Radius of Fillet, R | 76 mm |  |

**Table A3. UTS Reduced ANOVA**

| Source | SS | df | MS | F Value | P Value |  |
| --- | --- | --- | --- | --- | --- | --- |
| Model | 544.54 | 7 | 77.79 | 25.65 | < 0.0001 | significant |
| A-Temperature | 41.66 | 1 | 41.66 | 13.74 | 0.0009 |  |
| B-Waste Ratio | 22.21 | 1 | 22.21 | 7.32 | 0.0115 |  |
| AB | 144.75 | 1 | 144.75 | 47.73 | < 0.0001 |  |
| A² | 1.63 | 1 | 1.63 | 0.5380 | 0.4694 |  |
| B² | 23.45 | 1 | 23.45 | 7.73 | 0.0096 |  |
| A²B | 44.11 | 1 | 44.11 | 14.54 | 0.0007 |  |
| AB² | 11.74 | 1 | 11.74 | 3.87 | 0.0591 |  |
| Residual | 84.92 | 28 | 3.03 |  |  |  |
| Lack of Fit | 4.14 | 1 | 4.14 | 1.38 | 0.2496 | not significant |
| Pure Error | 80.78 | 27 | 2.99 |  |  |  |
| Cor Total | 629.46 | 35 |  |  |  |  |

**Table A4. Toughness Reduced ANOVA**

| Source | SS | df | MS | F Value | P Value |  |
| --- | --- | --- | --- | --- | --- | --- |
| Model | 5.05 | 8 | 0.6310 | 16.48 | < 0.0001 | significant |
| A-Temperature | 0.4434 | 1 | 0.4434 | 11.58 | 0.0021 |  |
| B-Waste Ratio | 1.17 | 1 | 1.17 | 30.66 | < 0.0001 |  |
| AB | 0.3998 | 1 | 0.3998 | 10.44 | 0.0032 |  |
| A² | 0.2393 | 1 | 0.2393 | 6.25 | 0.0188 |  |
| B² | 0.1001 | 1 | 0.1001 | 2.62 | 0.1175 |  |
| A²B | 0.4391 | 1 | 0.4391 | 11.47 | 0.0022 |  |
| AB² | 0.0002 | 1 | 0.0002 | 0.0061 | 0.9384 |  |
| A²B² | 0.2388 | 1 | 0.2388 | 6.24 | 0.0189 |  |
| Pure Error | 1.03 | 27 | 0.0383 |  |  |  |
| Cor Total | 6.08 | 35 |  |  |  |  |
| Model | 5.05 | 8 | 0.6310 | 16.48 | < 0.0001 | significant |

**Table A5. Stiffness Reduced ANOVA**

| Source | SS | df | MS | F Value | P Value |  |
| --- | --- | --- | --- | --- | --- | --- |
| Model | 8.820E+05 | 8 | 1.103E+05 | 18.39 | < 0.0001 | significant |
| A-Temperature | 45177.38 | 1 | 45177.38 | 7.54 | 0.0106 |  |
| B-Waste Ratio | 1.629E+05 | 1 | 1.629E+05 | 27.18 | < 0.0001 |  |
| AB | 95847.53 | 1 | 95847.53 | 15.99 | 0.0004 |  |
| A² | 56260.49 | 1 | 56260.49 | 9.39 | 0.0049 |  |
| B² | 1.239E+05 | 1 | 1.239E+05 | 20.67 | 0.0001 |  |
| A²B | 1.048E+05 | 1 | 1.048E+05 | 17.48 | 0.0003 |  |
| AB² | 37386.16 | 1 | 37386.16 | 6.24 | 0.0189 |  |
| A²B² | 60942.20 | 1 | 60942.20 | 10.17 | 0.0036 |  |
| Pure Error | 1.618E+05 | 27 | 5994.31 |  |  |  |
| Cor Total | 1.044E+06 | 35 |  |  |  |  |

**Table A6. Cost Reduced ANOVA**

| Source | SS | df | MS | F Value | P Value |  |
| --- | --- | --- | --- | --- | --- | --- |
| Model | 23110.40 | 8 | 2888.80 | 130.34 | < 0.0001 | significant |
| A-Temperature | 1.74 | 1 | 1.74 | 0.0784 | 0.7818 |  |
| B-Waste Ratio | 7654.57 | 1 | 7654.57 | 345.36 | < 0.0001 |  |
| AB | 90.49 | 1 | 90.49 | 4.08 | 0.0537 |  |
| A² | 282.13 | 1 | 282.13 | 12.73 | 0.0014 |  |
| B² | 219.52 | 1 | 219.52 | 9.90 | 0.0041 |  |
| A²B | 2.25 | 1 | 2.25 | 0.1015 | 0.7526 |  |
| AB² | 35.40 | 1 | 35.40 | 1.60 | 0.2175 |  |
| A²B² | 334.26 | 1 | 334.26 | 15.08 | 0.0006 |  |
| Pure Error | 576.27 | 26 | 22.16 |  |  |  |
| Cor Total | 23686.67 | 34 |  |  |  |  |

**APPENDIX B**


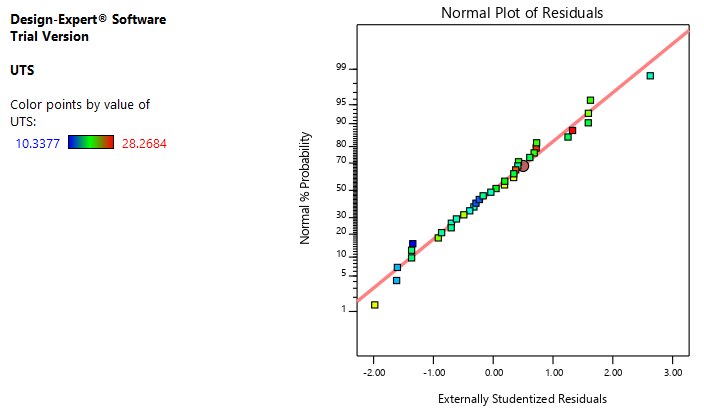


**Figure B1. Normal plot of residuals for UTS**


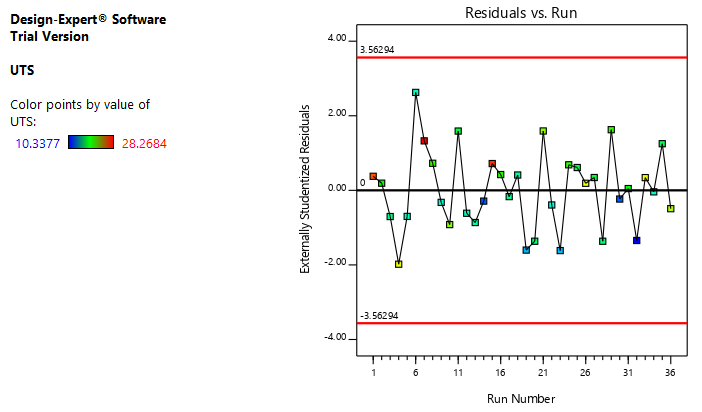


**Figure B2. Residual vs. Run of UTS Model**


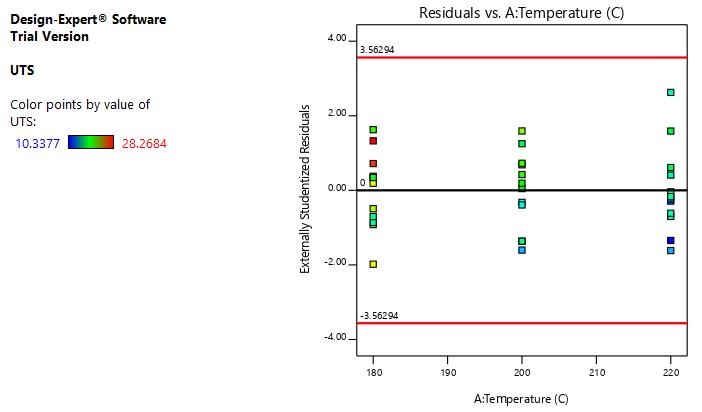

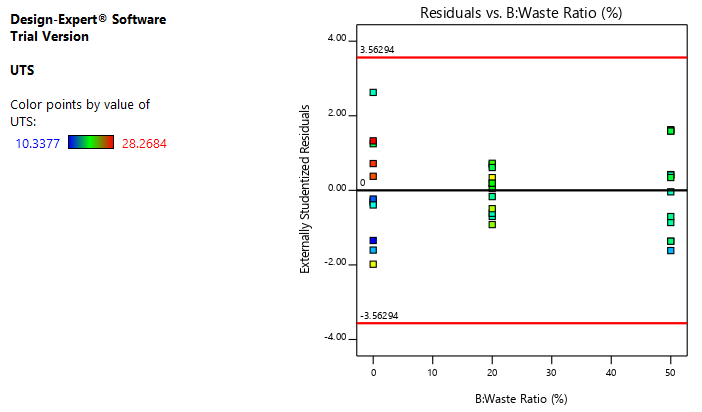


**Figure B3. Residuals vs. Temperature and Waste Ratio for UTS Model**
